# Effect of Oxygen Content on Current Stress-Induced Instability in Bottom-Gate Amorphous InGaZnO Thin-Film Transistors

**DOI:** 10.3390/ma12193149

**Published:** 2019-09-26

**Authors:** Sungju Choi, Jae-Young Kim, Hara Kang, Daehyun Ko, Jihyun Rhee, Sung-Jin Choi, Dong Myong Kim, Dae Hwan Kim

**Affiliations:** The School of Electrical Engineering, Kookmin University, Seoul 02707, Korea; sungjuchoi@kookmin.ac.kr (S.C.); kjp1486@kookmin.ac.kr (J.-Y.K.); hara3540@kookmin.ac.kr (H.K.); kdh9147@kookmin.ac.kr (D.K.); badagirs@kookmin.ac.kr (J.R.); sjchoiee@kookmin.ac.kr (S.-J.C.); dmkim@kookmin.ac.kr (D.M.K.)

**Keywords:** a-IGZO TFT, current stress, oxygen content, instability, electron trapping, oxygen flow rate, donor creation

## Abstract

The effect of oxygen content on current-stress-induced instability was investigated in bottom-gate amorphous InGaZnO (a-IGZO) thin-film transistors. The observed positive threshold voltage shift (ΔV_T_) was dominated by electron trapping in the gate insulator (GI), whereas it was compensated by donor creation in a-IGZO active regions when both current flows and a high lateral electric field were present. Stress-induced ΔV_T_ increased with increasing oxygen content irrespective of the type of stress because oxygen content influenced GI quality, i.e., higher density of GI electron traps, as well as typical direct current (DC) performance like threshold voltage, mobility, and subthreshold swing. It was also found that self-heating became another important mechanism, especially when the vertical electric field and channel current were the same, independent of the oxygen content. The increased ΔV_T_ with oxygen content under positive gate bias stress, positive gate and drain bias stress, and target current stress was consistently explained by considering a combination of the density of GI electron traps, electric field relaxation, and self-heating-assisted electron trapping.

## 1. Introduction

Since amorphous InGaZnO (a-IGZO) thin-film transistors (TFTs) are used in active-matrix organic light-emitting diode (AMOLED) television manufacturing, their reliability under long-term current stress is an important and challenging issue [1]. Although a-IGZO TFTs have been successfully commercialized into display products, next-generation displays with higher resolution, higher brightness, and longer product lifetime will demand more stable device characteristics. In particular, current stress-induced instability must be analyzed systematically along with various bias conditions, i.e., various gate-to-source voltage (V_GS_) and drain-to-source voltage (V_DS_) conditions, because current-driving TFTs in an AMOLED pixel as well as the TFTs in gate-driver circuitry experience various V_GS_ and V_DS_ conditions during real operation of the display circuits. On the other hand, the performance or stability of a-IGZO TFTs has been widely designed and optimized based on controlling the oxygen content (O-content) in IGZO thin films [2,3,4,5,6,7,8]. Therefore, thoroughly understanding the effect of O-content in IGZO thin films on current stress (CS)-induced instability under various V_GS_ and V_DS_ conditions is indispensable for the design of highly stable a-IGZO TFTs as well as for the design of current-driving schemes for high frame-rate display backplanes [9]. However, consolidated explanations of the effect of O-content on various CS-induced instabilities in a single framework are rare.

In this work, the effect of O-content on CS-induced instability is experimentally investigated in bottom-gate a-IGZO TFTs under various V_GS_ and V_DS_ conditions. To control the O-content in IGZO thin films, the oxygen flow rate (OFR) during the sputter deposition of the IGZO film is modulated. The OFR-dependent O-content is verified by X-ray photoelectron spectroscopy (XPS), which is consistent with previous studies [6,7,8]. Moreover, the measured current–voltage (I–V) and hysteresis characteristics as well as the experimentally extracted subgap density of states (DOS) in a-IGZO active films are combined. Self-heating and power consumption are considered by using technology computer-aided design (TCAD) simulation. The influence of O-content on the CS-instability in IGZO TFTs under various V_GS_/V_DS_ conditions is elucidated from material science and device physics perspectives.

## 2. Experimental Procedure and Material Properties

The fabrication of a-IGZO TFTs with a bottom-gate structure is described as follows. The first room temperature (RT) sputtered deposition of a-IGZO on a glass substrate and patterning of the molybdenum (Mo) gate were followed by plasma-enhanced chemical vapor deposition (PECVD) of SiN_X_ and SiO_2_ at 370 °C, which acted as a gate dielectric (the equivalent oxide thickness, T_ox_, was 258 nm). A 50 nm-thick a-IGZO thin film was then deposited by direct current (DC) sputtering (3 kW) at room temperature (RT) in a mixed atmosphere of Ar (35 sccm) and O_2_ by modulating the OFR to produce O-poor (21 sccm), O-mid (42 sccm), and O-rich (63 sccm) TFT devices. Next, an etch stopper (SiO_X_) layer was deposited by PECVD at 150 °C. For the formation of the source/drain (S/D) electrodes, Mo was sputtered at RT. A passivation layer (SiO_X_ and SiN_X_; each 100 nm thick) was subsequently deposited. Finally, the devices were annealed at 250 °C for 1 h in a furnace. The width (W_ch_) and length of the device channel (L_ch_) were 200 and 100 μm, respectively.

**Figure 1:**
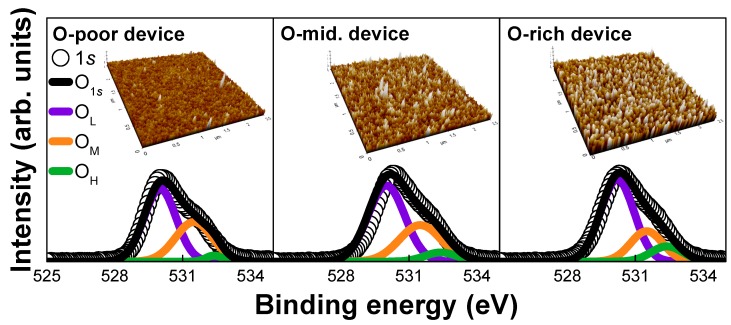
X-ray photoelectron spectroscopy (XPS) *O_*1*s_* spectra of InGaZnO (IGZO) films upon varying the flow rate in a mixed atmosphere of Ar and O_2_ (oxygen flow rate (OFR) = 35 sccm). The OFR is modulated to produce O-poor (OFR = 21 sccm), O-mid (42 sccm), and O-rich (63 sccm) IGZO thin films. The inset shows the surface morphology of IGZO thin films as a function of OFR, as observed by atomic force microscopy (AFM).

To check if the OFR modulated the O-content in IGZO active films, the XPS spectra of the devices were analyzed. Figure 1 shows the *O_*1*s_* spectra of the IGZO films. The low-binding-energy component (*O_L_*) located at 530 eV is usually attributed to O_2_^−^ ions surrounded by Zn, Ga, and in atoms in the IGZO compound system. The middle binding-energy component (*O_M_*) centered at 531.5 eV is associated with O_2_^−^ ions that are in the oxygen-deficient regions of the IGZO matrix. Therefore, a change in the peak area ratio is related to the concentration of oxygen vacancies. O_M_-related oxygen vacancies supply free electron carriers in the IGZO film, resulting in an increase of electron concentration. The high binding-energy component (*O_H_*) located at 532.4 eV is mainly attributed to the presence of loosely bound, chemisorbed oxygen impurities (−CO_3_^−^ adsorbed H_2_O or adsorbed O_2_^−^) on the surface of the film. The observed *O_*1*s_* peak can be deconvoluted into three peaks, namely, *O_L_*, *O_M_*, and *O_H_*, as shown in Equation (1), and their characteristic parameters are summarized in Table 1:

(1) $$O_{1s}=O_{L}+O_{M}+O_{H}=N_{L}\cdot \exp\left(-{\left(\frac{E-E_{L}}{kT_{L}}\right)}^{2}\right)+N_{M}\cdot \exp\left(-{\left(\frac{E-E_{M}}{kT_{M}}\right)}^{2}\right)+N_{H}\cdot \exp\left(-{\left(\frac{E-E_{H}}{kT_{H}}\right)}^{2}\right)$$

It is clearly observed in Figure 1 and Table 1 that *O_M_* decreases (from 33% to 28.5%) and *O_H_* increases (from 5% to 11%) as the OFR increases. The O-content is proportional to the OFR trend. The results of the XPS analysis suggest that the oxygen content in the IGZO film can be controlled by modulating the OFR during sputter deposition of the IGZO thin film. This is consistent with previous reports [6,7,8].

The surface roughness of the IGZO films was characterized by using atomic force microscopy (AFM, Suwon, Republic of Korea), the results of which are presented in the inset of Figure 1. The measured roughness values for O-poor, O-mid, and O-rich devices were 0.236, 0.386, and 0.562 nm, respectively. As the O-content becomes larger with increasing OFR, the IGZO surface becomes rougher.

The I–V characteristics of the IGZO TFTs were measured using an HP4156C (Keysight, Santa Rosa, CA, USA) semiconductor parameter analyzer at RT in the dark. The capacitance–voltage (C–V) characteristics were measured between the gate and the source tied to drain terminals by using an HP4294 LCR meter (Keysight, Santa Rosa, CA, USA). The subgap DOS was extracted using multi-frequency C–V spectroscopy [10], wherein frequencies of 2 kHz, 100 kHz, and 1 MHz were used with a ramp-up rate of 0.4 V/s and a small signal of 0.1 mV.

In terms of the V_GS_ and V_DS_ conditions under CS, three stress conditions were used during a stress time of 10^4^ s: positive gate bias stress (PGBS), where V_GS_/V_DS_ = 20/0 V; positive gate and drain bias stress (PGDBS), where V_GS_/V_DS_ = 20/10 V regardless of the OFR; and target current stress (TCS) with 20 μA for various O-contents, where V_GS_/V_DS_ = 20/10 V for O-poor, 20.5/11 V for O-mid, and 22/12 V for O-rich. PGBS and PGDBS were investigated first, after which the TCS was analyzed. Here, TCS means that the V_GS_/V_DS_ condition varied depending on the O-content, where V_GS_ is determined so that the value of overdrive voltage (V_GS_ − V_T_) remains the same according to the O-content-dependent threshold voltage (V_T_). V_DS_ is then determined such that the drain-to-source current (I_DS_) is maintained at the same value (20 μA) as that of the O-poor TFT at V_GS_/V_DS_ = 20/10 V. Therefore, our TCS condition establishes both constant vertical electric field and constant current, at least in pristine states before CS.

## 3. Results and Discussion

**Figure 2:**
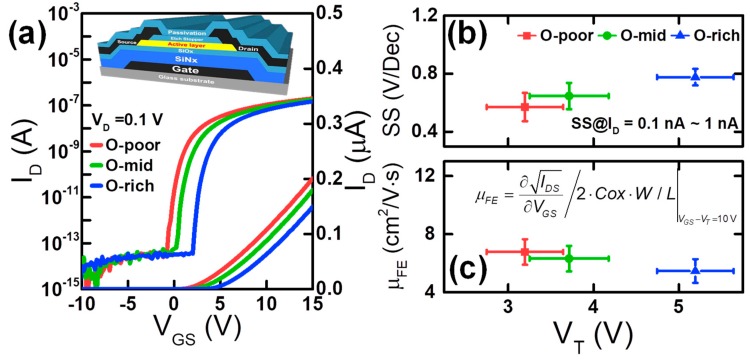
(**a**) I_D_*–*V_GS_ transfer curve characteristic at V_DS_ = 0.1 V for various O-contents. The inset is the fabricated a-IGZO thin-film transistor (TFT) with an inverted staggered bottom-gate structure. The statistical data of (**b**) subthreshold swing (SS)-V_T_, and (**c**) μ_FE-_V_T_ were extracted from 10 devices for each O-content.

The O-content-dependent parameters of pristine TFT devices, such as I–V, V_T_, subthreshold swing (SS), and saturation field-effect mobility (μ_FE_), are shown in Figure 2a–c, where V_T_ was extracted by linear extrapolation. SS was determined from 10^−10^ to 10^−9^ A in the subthreshold region. In addition, μ_FE_ was extracted using the square root method at V_GS_ − V_T_ = 10 V and V_DS_ = 10 V. With increasing OFR, i.e., with higher O-content, V_T_ and SS increase, and μ_FE_ decreases, as shown Figure 2b,c. These results indicate that oxygen-related defects near the conduction band minimum (E_C_) increased with increasing OFR [11]. Therefore, the subgap DOS should be investigated to understand the effect of O-content on TFT performance and stability.

The extracted DOS (*g*(*E*)) values near the E_C_ were divided into three components according to their energy level distribution: 1. shallow donor-like states, characteristic, and center energies of the Gaussian peaks (*N_SD_*, *kT_SD_,* and *E_SD_*), 2. acceptor-like deep states and characteristic *N_DA_* and *kT_DA_*, and 3. tail states and characteristic *N_TA_* and *kT_TA_*. The extracted *g*(E) value near *E_C_* was modeled according to Equation (2):

(2) $$g\left(E\right)=g_{TA}\left(E\right)+g_{DA}\left(E\right)+g_{SD}\left(E\right)=N_{TA}\exp\left(-\frac{E_{C}-E}{kT_{TA}}\right)+N_{DA}\exp\left(-\frac{E_{C}-E}{kT_{DA}}\right)+N_{SD}\exp\left(-{\left(\frac{E_{C}-E-E_{SD}}{kT_{SD}}\right)}^{2}\right)$$

**Figure 3:**
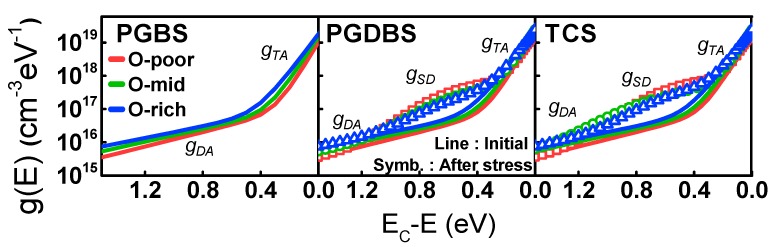
Density of states (DOS) over a range of O-content as stress time under positive gate bias stress (PGBS), positive gate and drain bias stress (PGDBS), and target current stress (TCS). Lines denote the initial state, and symbols indicate values after each stress condition.

The extracted DOS and their parameters are summarized in Figure 3 and Table 2. They were extracted for the initial state and after PGBS, PGDBS, and TCS.

The effects of O-content on V_T_, SS, and μ_FE_ (Figure 2b,c) can be explained by considering both the XPS and AFM results (Figure 1) and the DOS (Figure 3). At first, V_T_ increases as the O-content increases. This is explained by the decrease in the number of oxygen vacancies (V_O_s) in the TFT devices with larger O-content, as shown in the XPS spectra in Figure 1. V_O_s are well-known shallow donors [12,13]. Second, SS increases with increasing O-content, which is explained by the higher *g_TA_*(*E*) and *g_DA_*(*E*) in the TFT devices with more O-content, as shown in Figure 3. Higher *g_TA_*(*E*) and *g_DA_*(*E*) result from an ion bombardment process during the sputter deposition with the increase of OFR [11]. Finally, μ_FE_ decreases as the O-content increased, which can be explained as follows. In amorphous multi-metal oxides, some potential barriers are inherently present between neighboring ions at the E_C_ and affect the electrons. Such barriers hinder electron transport and lower the mobility because of the different metal ion radii that result from the non-uniform overlap of conduction electron orbitals among In–O, Ga–O, and Zn–O bonds [14]. Subsequently, the percolation barrier height is effectively lowered because of the higher Fermi energy (E_F_) in lower O-content or higher V_GS_ conditions, thus leading to higher μ_FE_. Moreover, the AFM topography of the sample shown in Figure 1 suggests that either the surface roughness scattering or trap density in IGZO increases for higher O-content, which is another reason for lower μ_FE_.

It is usually reported that the electrical performance in IGZO TFTs is significantly affected by trap sites near the E_C_ [11]. Several key parameters such as surface roughness, g_TA_(E), g_SD_(E), and g_DA_(E) around the E_C_ are both energetically and locally distributed throughout the energy bandgap with energy level dependency, causing carrier trapping and emission in each energy state (i.e., surface roughness scattering, multiple trapping, subgap hopping process, and thermal release events). Therefore, high trap density and high surface roughness decrease the mean free path of carriers and the ability to accumulate carriers, thus leading to the degradation of μ_FE_ and SS.

**Figure 4:**
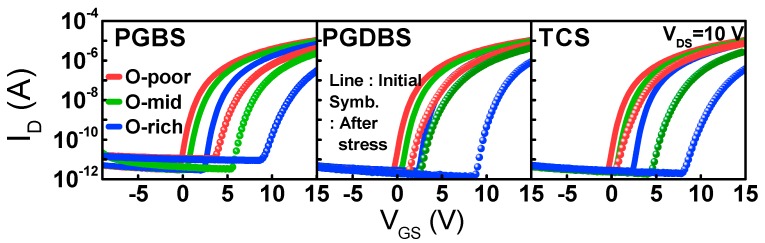
Transfer V_GS_–I_DS_ characteristics at V_DS_ = 10 (V) over a range of O-content as stress time under PGBS, PGDBS, and TCS. Lines denote the initial state, and symbols indicate values after each stress condition.

**Figure 5:**
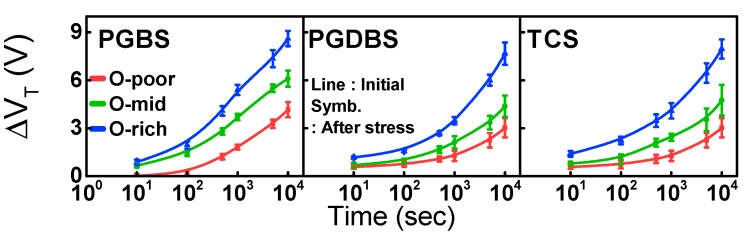
ΔV_T_ for various O-contents as stress time under PGBS, PGDBS, and TCS.

The I_DS_–V_GS_ transfer characteristics before and after stress are shown in Figure 4. The CS-induced V_T_ shift (ΔV_T_), which increased with O-content irrespective of the type of stress, is summarized in Figure 5. To clarify the degradation that occurred only in the a-IGZO active layer, g(E) values before and after CS were also compared, as shown in Figure 3 and Table 2. Under the influence of PGBS and/or PGDBS, V_T_ of the TFTs frequently shifted in the positive direction. The physical origin of PBTS instability has been classified largely by either electron trapping (e-trapping) into the gate insulator (GI) interface [15,16] or the change in defect states in the active region [17]. In the former case, e-trapping becomes more accelerated with increasing temperature and/or electric field [18] as well as high density of electron traps in the gate insulator bulk interface. In the latter case, donor creation followed by a negative ΔV_T_ occurs, especially in short-channel TFTs [19,20], whereas oxygen–dimer bond breaking was very recently observed as the physical origin of positive ΔV_T_ [21]. Furthermore, in comparison with PGBS, ΔV_T_ under PGDBS and/or TCS is very complicated because of various combinations of V_GS_ and V_DS_, and competition between e-trapping and donor creation has been reported [22].

Only under PGDBS and TCS, i.e., when I_DS_ flowed through the channel, were shallow donor states consistently created below the E_C_, as shown in Figure 3 and Table 2. The physical origin of donor creation is still controversial. In this study, it seemed to be dominated by V_O_ ionization [19] or by peroxide formation [23]. Under PGDBS or TCS, electrons gained enough energy from the lateral field and were able to break the weakest oxygen bond or generate holes. In the former case, V_O_s that are not filled by electrons are generated by structure relaxation and are doubly positively charged (V_O_^2+^) [22]. In the latter case, hole-intermediated peroxide generation occurs, i.e., O^2−^ + O^2−^ → O_2_^2−^ + 2e^−^. In both cases, donor creation lowers V_T_ when stress is applied. This is contradictory to the observed positive ΔV_T_. Therefore, the dominant mechanism behind the positive ΔV_T_ compensates donor creation (origin of a negative ΔV_T_), thus leading to eventual positive ΔV_T_. It can be assumed that the positive ΔV_T_ during stress originates from the e-trapping in the GI.

**Figure 6:**
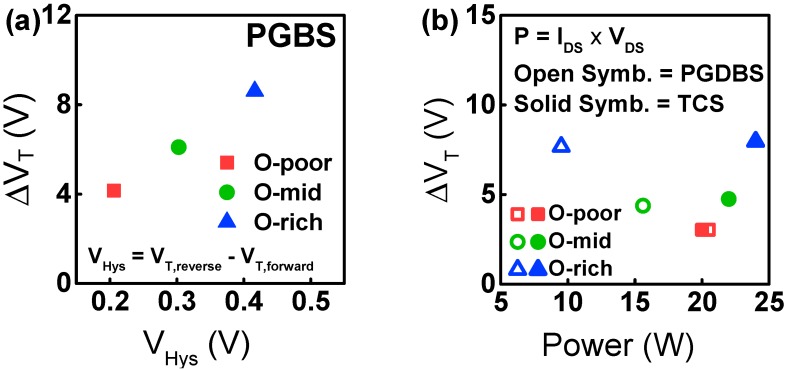
The OFR-dependent relationships (**a**) between ΔV_T_ and V_Hys_ and (**b**) between ΔV_T_ and the average power consumption under PGDBS and TCS. Closed symbols denote PGDBS conditions, and open symbols indicate TCS conditions.

To validate our assumption, we examined the relationship between hysteresis and ΔV_T_, as shown in Figure 6a. The hysteresis effect is known to change V_T_ when V_GS_ is swept from negative to positive values and then the transfer curve is measured again while sweeping from positive to negative V_GS_. That is, after accumulated carriers are trapped in shallow or deep traps of the GI, the captured carriers cause a screening effect to the V_GS_. Therefore, the hysteresis voltage (V_Hys_) is a good indicator of the quality of the GI as well as the GI/IGZO interface. Here, V_Hys_ is defined as the difference in V_T_ between double V_GS_ sweeps, i.e., the sweep from 20 to −20 V and that from −20 to 20 V as denoted in Figure 6a. Figure 6a indicates that the oxygen-content-dependent, PGBS-induced ΔV_T_ is well correlated with the V_Hys_ depending on the O-content, which validates the assumption that the PGBS/PGDBS/TCS-induced instability is dominated by e-trapping in the GI and/or interface.

**Figure 7:**
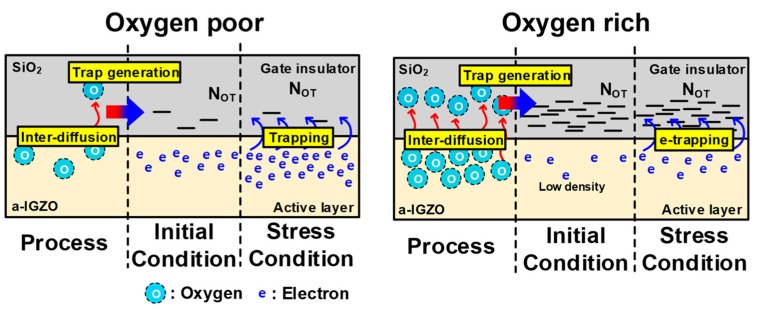
Production of electron trap centers according to O-content.

The effect of O-content on ΔV_T_ and V_Hys_ can be explained as follows. As the O-content in the a-IGZO active layer increases, a larger number of oxygen atoms at the interface or in the active layer diffuse into the SiO_2_ of GI owing to the stronger Si–O affinity in comparison to that of Ga–O, Zn–O, or In–O (Si–O > Ga–O > Zn–O > In–O) [24] during fabrication. Increased oxygen interstitials generate electron traps in the GI [25], which is consistent with relatively poor GI quality (i.e., larger ΔV_T_) and hysteresis in O-rich devices, as shown Figure 7. Here, N_OT_ symbolizes the spatial density of electron traps in the GI.

Given the O-content of the active layer, the positive ΔV_T_ increased in the following order: PGDBS < TCS < PGBS, as shown in Figure 5. In the PGBS condition, only e-trapping in the GI occurred effectively, both in the source and drain regions. However, in the PGDBS and TCS conditions, e-trapping near the drain was significantly alleviated, owing to the release of the vertical electric field and creation of donor states. Therefore, the largest positive ΔV_T_ occurred for PGBS conditions.

In addition, it should be noted that, for a given O-content, ΔV_T_ under TCS is larger than that under PGDBS. This can be explained by the self-heating assisted e-trapping [26,27,28], vertical electric field, and bulk oxide trap. To analyze the Joule heating effect under stress conditions, the average power consumption was calculated by multiplying the drain current by V_DS_ under stress conditions, as shown in Figure 6b. Joule heating under TCS was greater than that under PGDBS. Such Joule heating accumulated from self-heating in the active layer as the thermal conductivity of a-IGZO is quite low [29]. E-trapping can then be activated to a greater extent via thermionic field emission-assisted GI trapping. Under PGDBS, although the O-poor device generated more heat in the active layer than that in the O-mid and O-rich devices, the ΔV_T_ of the O-rich device was the largest. These results indicate that e-trapping is dominated by the N_OT_ rather than by the self-heating effect. Therefore, GI quality is the key factor for determining ΔV_T_ under TCS, PGDBS, and PGBS conditions for various O-contents. Self-heating-assisted e-trapping also becomes more significant for devices with the same O-content as the CS dissipates more Joule heating and power.

**Figure 8:**
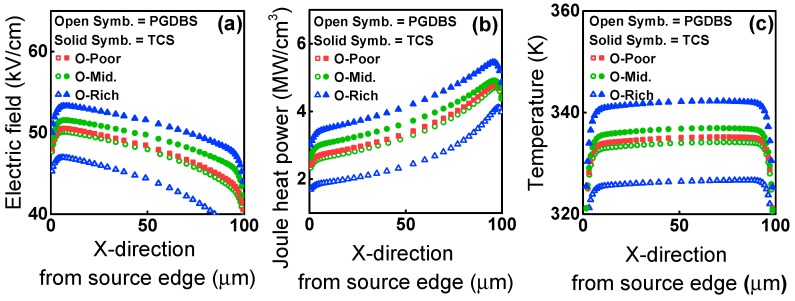
The simulated (**a**) electric field, (**b**) Joule heat power, and (**c**) temperature of the device based on the configuration of V_GS_/V_DS_.

To validate our discussion, a well-calibrated TCAD simulation was performed by incorporating the energy band structure, a DOS model depending on the O-content, trap-limited conduction, and a self-heating model into Silvaco ATLAS-2D [30]. The electric field, Joule heating power, and device temperature were simulated under different conditions, as shown in Figure 8a–c. As shown in Figure 8c, regardless of the O-content, self-heating was more significant in TCS than in PGDBS. Therefore, as shown in Figure 6b, the ΔV_T_ in TCS is larger than that in PGDBS irrespective of O-content. Here, it should be noted that, in the O-poor device, the TCS condition is the same as the PGDBS condition. Eventually, the CS-induced instability is determined by the increasing N_OT_ with O-content and the ΔV_T_-compensation by donor creation. Specifically, under TCS, the increasing self-heating with O-content becomes another key factor, although both the initial vertical electric field and initial I_DS_ are the same, independent of the O-content.

Our results suggest that the effect of O-content on CS-induced instability can be explained consistently for all cases considered in this study, i.e., TCS, PGDBS, and PGBS conditions.

## 4. Conclusions

The effect of O-content on CS-induced instability is investigated in bottom-gate a-IGZO TFTs. Under PGDBS and TCS conditions, donor creation was clearly observed in a-IGZO active regions. The observed positive ΔV_T_ was dominated by e-trapping in the GI. Because the O-content affects the GI quality as well as the typical DC-performance parameters, such as V_T_, SS, and μ_FE_, stress-induced ΔV_T_ increased with increasing O-content irrespective of the type of stress. For specific O-content, ΔV_T_ increased in the following order: PGDBS < TCS < PGBS, which can be explained by considering a combination of electric field relaxation via donor creation and self-heating-assisted e-trapping. Our results suggest that careful joint optimization of the GI and active layer is indispensable for designing highly stable a-IGZO TFTs. The detailed findings are useful for designing stable current-driving schemes for compensating electrical degradation and for circuits in high-frame-rate displays.

## Figures and Tables

**Table 1:** Extracted XPS parameters (initial).

O-Content		O-Poor	O-Medium	O-Rich
*O_L_*	*N_L_* (A.U)	1.20 × 10^5^	1.25 × 10^5^	1.30 × 10^5^
	*KT_L_* (eV)	1		
	*E_L_* (eV)	530		
*O_M_*	*N_M_* (A.U.)	0.65 × 10^5^	0.60 × 10^5^	0.50 × 10^5^
	*KT_M_* (eV)	1.15	1.1	1.1
	*E_M_* (eV)	531.5		
*O_H_*	*N_H_* (A.U.)	0.10 × 10^5^	0.2 × 10^5^	0.25 × 10^5^
	*KT_H_* (eV)	0.5	0.75	1
	*E_H_* (eV)	532.4		

**Table 2:** Extracted DOS parameters (initial and after PGBS, PGDBS, and TCS).

Parameter	O-Content	Stress Condition	*N_dos_* (eV^−1^ cm^−3^)	*kT_dos_* (eV)	*E_dos_* (eV)
*g_TA_* (E)	O-poor	All conditions	2.0 × 10^19^	0.06	–
	O-mid	All conditions	2.3 × 10^19^	0.065	–
	O-rich	All conditions	3.0 × 10^19^	0.075	–
*g_DA_* (E)	O-poor	All conditions	1.5 × 10^17^	0.4	–
	O-mid	All conditions	1.6 × 10^17^	0.47	–
	O-rich	All conditions	1.9 × 10^17^	0.5	–
*g_SD_* (E)	O-poor	Initial	–	0.4–0.45	0.3
		After PGBS	–		
		After PGDBS	5 × 10^17^		
		After TCS	6 × 10^17^		
	O-mid	Initial	–		
		After PGBS	–		
		After PGDBS	3 × 10^17^		
		After TCS	3 × 10^17^		
	O-rich	Initial	–		
		After PGBS	–		
		After PGDBS	2 × 10^17^		
		After TCS	3 × 10^17^		
